# Glasdegib plus intensive or non-intensive chemotherapy for untreated acute myeloid leukemia: results from the randomized, phase 3 BRIGHT AML 1019 trial

**DOI:** 10.1038/s41375-023-02001-z

**Published:** 2023-08-21

**Authors:** Mikkael A. Sekeres, Pau Montesinos, Jan Novak, Jianxiang Wang, Deepa Jeyakumar, Benjamin Tomlinson, Jiri Mayer, Erin Jou, Tadeusz Robak, David C. Taussig, Hervé Dombret, Akil Merchant, Naveed Shaik, Thomas O’Brien, Whijae Roh, Xueli Liu, Wendy Ma, Christine G. DiRienzo, Geoffrey Chan, Jorge E. Cortes

**Affiliations:** 1 Division of Hematology, Sylvester Comprehensive Cancer Center, University of Miami, Miami, FL USA; 2 Hospital Universitari i Politècnic La Fe, Valencia, Spain; 3 CIBERONC, Instituto Carlos III, Madrid, Spain; 4 Department of Internal Medicine and Hematology, University Hospital Kralovske Vinohrady, Prague, Czech Republic; 5 Department of Haematology, 3rd Faculty of Medicine, Charles University and Faculty Hospital Kralovske Vinohrady, Prague, Czech Republic; 6 Department of Clinical Hematology, Institute of Hematology and Blood Diseases Hospital, Tianjin, China; 7 University of California, Irvine, Chao Family Comprehensive Cancer Center, Orange, CA USA; 8 Division of Hematology, University Hospitals of Cleveland Seidman Cancer Center, Case Western Reserve University, Cleveland, OH USA; 9 Department of Internal Medicine Hematology and Oncology, University Hospital Brno and Masaryk University, Brno, Czech Republic; 10 Department of Hematology/Oncology, Donald and Barbara Zucker School of Medicine at Hofstra/Northwell, Manhasset, NY USA; 11 Department of Hematology, Medical University of Lodz, Lodz, Poland; 12 The Royal Marsden Hospital, London, UK; 13 Institut de Recherche Saint-Louis, Hôpital Saint-Louis Assistance Publique-Hôpitaux de Paris, Université Paris Cité, Paris, France; 14 Division of Hematology and Cellular Therapy, Cedars Sinai Cancer, Los Angeles, CA USA; 15 Pfizer Oncology, Pfizer Inc, San Diego, CA USA; 16 Pfizer Inc, Collegeville, PA USA; 17 Georgia Cancer Center, Augusta, GA USA

**Keywords:** Targeted therapies, Targeted therapies

## Abstract

This is the primary report of the randomized, placebo-controlled phase 3 BRIGHT AML 1019 clinical trial of glasdegib in combination with intensive chemotherapy (cytarabine and daunorubicin) or non-intensive chemotherapy (azacitidine) in patients with untreated acute myeloid leukemia. Overall survival (primary endpoint) was similar between the glasdegib and placebo arms in the intensive (*n* = 404; hazard ratio [HR] 1.05; 95% confidence interval [CI]: 0.782–1.408; two-sided *p* = 0.749) and non-intensive (*n* = 325; HR 0.99; 95% CI: 0.768–1.289; two-sided *p* = 0.969) studies. The proportion of patients who experienced treatment-emergent adverse events was similar for glasdegib versus placebo (intensive: 99.0% vs. 98.5%; non-intensive: 99.4% vs. 98.8%). The most common treatment-emergent adverse events were nausea, febrile neutropenia, and anemia in the intensive study and anemia, constipation, and nausea in the non-intensive study. The addition of glasdegib to either cytarabine and daunorubicin or azacitidine did not significantly improve overall survival and the primary efficacy endpoint for the BRIGHT AML 1019 phase 3 trial was not met. Clinical trial registration**:** ClinicalTrials.gov: NCT03416179.

## Introduction

Acute myeloid leukemia (AML) is an aggressive hematologic malignancy most commonly diagnosed in patients aged 65–74 years [1]. Despite recent advances in therapy, the 5-year overall survival (OS) rate remains low (typically <40%) [1, 2]. The decision whether to receive intensive or non-intensive therapy incorporates relative risks of age, comorbidities, performance status, disease biology, and patient goals, as well as other considerations such as regional availability of drugs [3–6].

Intensive (7 + 3) chemotherapy usually includes a cytarabine and anthracycline backbone [7, 8]; however, this approach may be too toxic for patients with advanced age or comorbidities and is less effective in those with adverse genomic features and increased treatment resistance [3, 9]. For patients unable to undergo intensive treatment, effective but less-intensive options include low-dose cytarabine (LDAC) and hypomethylating agent-based regimens and palliative care [8–10]. For many, complete remission (CR) rates can be low ( < 30%) with median OS often <1 year [11–15]. To address their unmet needs, new combination therapy approaches have been explored, including adding venetoclax or targeted isocitrate dehydrogenase inhibitors to hypomethylating agents [16–18].

The Hedgehog signaling pathway is critically involved in embryonic patterning and organ development, including hematopoiesis [19]. Its activation has been mechanistically linked to the development and progression of solid and hematologic malignancies, including AML [19]. Glasdegib is a potent, oral inhibitor of the Hedgehog signaling pathway through the inhibition of Smoothened [20]. Glasdegib in combination with LDAC is approved for the treatment of newly diagnosed AML in adults aged ≥75 years or with comorbidities that preclude use of intensive induction chemotherapy in the United States [21], and in adults who are not candidates for standard induction chemotherapy in Europe [22]. Glasdegib was approved following positive results of the phase 2 BRIGHT AML 1003 trial, wherein glasdegib in combination with LDAC versus LDAC alone significantly increased OS and response rates in patients with newly diagnosed AML or myelodysplastic syndrome (MDS) who were ineligible for intensive chemotherapy [23]. We report the efficacy, safety, and pharmacokinetic results of the placebo-controlled phase 3 BRIGHT AML 1019 clinical trial of glasdegib in combination with intensive (cytarabine and daunorubicin) or non-intensive (azacitidine) chemotherapy in patients with untreated AML.

## Methods

### Study design

BRIGHT AML 1019 comprised two global, double-blind, phase 3 studies (NCT03416179). Details of the study design, including full eligibility criteria, were previously published [24]. Briefly, BRIGHT AML 1019 included an intensive and a non-intensive study. In both studies, patients were randomized 1:1 to placebo or glasdegib. The intensive study evaluated glasdegib or placebo plus cytarabine and daunorubicin; the non-intensive study evaluated glasdegib or placebo plus azacitidine. Patients’ study assignment was decided by the investigator based on assessment of the patient’s fitness and goals of their care. Recruitment occurred from April 2018 to January 2020 and follow-up continued until December 2022.

Adults were eligible if they had untreated AML (defined according to the World Health Organization 2016 Classification [25]) with adequate organ function and QTc interval ≤470 ms. All anti-cancer treatments must have been discontinued ≥2 weeks from study entry. At randomization, patients in the intensive study were stratified by genetic risk (favorable vs. intermediate vs. adverse, by 2017 European LeukemiaNet [ELN] categories [10]), and age ( ≤ 60 vs. >60 years). Patients in the non-intensive study were similarly stratified by genetic risk and by age ( < 75 vs. ≥75 years).

The study complied with the Declaration of Helsinki and the International Conference on Harmonization Good Clinical Practice guidelines. The protocol was approved by the institutional review boards of the participating institutions, and all patients provided signed informed consent.

### Study treatment and procedures

Patients in the intensive study received glasdegib 100 mg once daily or placebo starting on Day 1 and continuing for up to 2 years or until treatment failure, hematologic relapse, disease progression, unacceptable toxicity, measurable residual disease (MRD)-negative post-consolidation and/or hematopoietic stem cell transplantation (HSCT), consent withdrawal, or death. ‘7 + 3’ induction therapy consisted of intravenous cytarabine 100 mg/m^2^ for 7 days and daunorubicin 60 mg/m^2^ for 3 days. A second induction could be given if the patient had not achieved remission after the first cycle or at the investigator’s discretion. The second induction could be either a 7 + 3 or 5 + 2 schedule according to investigator’s choice. Consolidation consisted of intravenous cytarabine 1 g/m^2^ (patients aged ≥60 years) or 3 g/m^2^ (patients aged <60 years) administered twice daily on Days 1, 3, and 5 of 28-day cycles for up to four cycles; alternative dosing schedules could be used per local prescribing information. Glasdegib or placebo could be continued regardless of chemotherapy dose modifications or delays.

Patients in the non-intensive study received glasdegib 100 mg once daily or placebo, starting on Day 1, plus subcutaneous or intravenous azacitidine 75 mg/m^2^ for 7 days in 28-day cycles. Treatment continued for at least six cycles or until unacceptable toxicity, disease progression, consent withdrawal, or death.

In both studies, patients proceeding to HSCT interrupted study therapy 28 days before the start of the conditioning regimen. Single-agent, blinded glasdegib/placebo therapy could be resumed 30–60 days post-HSCT assuming absolute neutrophil count engraftment, no ongoing grade ≥2 graft-versus-host disease, and no ongoing serious adverse events (AEs).

For the intensive study, disease assessments were performed after the completion of the first induction chemotherapy, at the end of induction upon at least partial hematologic recovery, at the end of the consolidation period, and before and after HSCT. In addition, patients in remission received disease assessments annually or when relapse was suspected. For the non-intensive study, disease assessments were performed when CR was suspected or after Cycle 6 (whichever was earlier), pre-HSCT, annually for patients in remission, and whenever progressive disease was suspected.

### Endpoints

The primary endpoint of both studies was OS, defined as time from randomization to death from any cause. Patients were not censored at HSCT as HSCT was part of the treatment plan, given that the patient was eligible. Secondary efficacy endpoints included response rates and time-to-treatment response (non-intensive study only). Disease response was evaluated using the 2017 ELN recommendations for diagnosis and management of AML in adults [10] and as defined previously [24]. Time-to-treatment response was defined as time from randomization to CR/CR with incomplete hematologic recovery (CRi) or CR/CR with partial hematologic recovery (CRh). Patient-reported fatigue, measured at Week 8 (intensive) and Week 12 (non-intensive) by the MD Anderson Symptom Inventory AML/MDS Module (MDASI-AML/MDS), was a key secondary efficacy endpoint. Other endpoints included safety, pharmacokinetics, and patient-reported outcomes. Bone marrow and blood biomarkers were exploratory endpoints. Additional details can be found in Supplementary Information.

### Statistical analysis

The primary objective was to demonstrate that glasdegib combined with cytarabine and daunorubicin (intensive study) or azacitidine (non-intensive study) is superior to placebo in prolonging OS in patients with untreated AML. Efficacy was assessed in all randomized patients (full analysis set). Safety was assessed in all patients who received at least one dose of study drug. Pharmacokinetic and biomarker data were analyzed in relevant patient sets as described in the study protocol [24]. OS was estimated using the Kaplan–Meier method. Hazard ratios (HR) and corresponding 95% confidence intervals (CI) were determined using the Cox proportional hazards model. The proportions of patients achieving each disease-specific efficacy endpoint (CR, CRh, CRi, morphologic leukemia-free state, and partial remission [PR]) were estimated with two-sided 95% CI using the exact method, and the proportions achieving each endpoint were compared between arms using the Cochran-Mantel-Haenszel stratified test. The proportion of responders on the patient-reported fatigue item of the MDASI-AML/MDS questionnaire was estimated with two-sided 95% CI using normal approximation and compared between arms using the Cochran-Mantel-Haenszel stratified test. Safety, pharmacokinetics, and time to clinical response were summarized descriptively. *P-* values were not adjusted for multiple comparisons.

As previously described [24], the planned sample size for the intensive study was 400 patients; 400 patients with 267 deaths would provide 90% power to detect an improvement in OS with HR = 0.67 (translated from a median OS of 21.0 vs. 31.5 months) using a one-sided log-rank test at a significance level of 0.025 and a three-look group-sequential design. A composite median OS of 21 months was based on prior studies that reported a median OS of 23.7 months in adults aged 17–60 years with AML treated with intensive chemotherapy, and a median OS of 15 months in adults >60 years of age [26, 27].

The planned sample size for the non-intensive study was 320 patients; 320 patients with 220 deaths would provide 90% power to detect an improvement in OS with HR = 0.64 (translated from a median OS of 10.4 months vs. 16.2 months) using a one-sided log-rank test at a significance level of 0.025 and a two-look group-sequential design. The median OS of 10.4 months was based on a study of patients ≥65 years of age with untreated AML and >30% bone marrow blasts [15].

## Results

### Patients and treatment

**Fig. 1:**
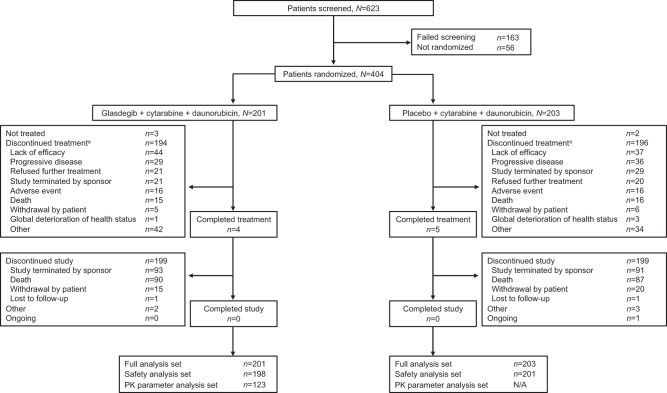
Patient disposition in the intensive study. ^a^With glasdegib or placebo. N/A not applicable, PK pharmacokinetic.

**Fig. 2:**
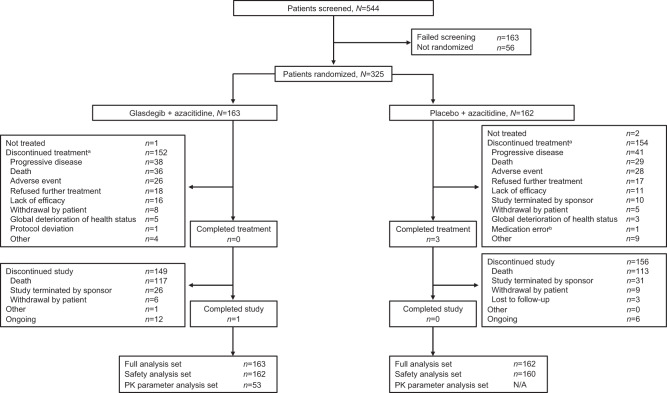
Patient disposition in the non-intensive study. ^a^With glasdegib or placebo; ^b^Without an associated adverse event. N/A not applicable, PK pharmacokinetic.

A total of 729 patients were enrolled in the studies; 404 patients were randomized and 399 received treatment in the intensive study, and 325 were randomized and 322 received treatment in the non-intensive study (Figs. 1, 2). In both studies, proportionately more males than females were assigned to receive glasdegib than placebo (Table 1). Patients in the non-intensive study versus the intensive study were older (median age 73 vs. 59 years) with worse Eastern Cooperative Oncology Group performance status (26.5% vs. 8.9% had performance status ≥2). Baseline genetic abnormalities are presented in Table S1.

**Table 1:** Baseline demographics and clinical characteristics of patients treated with glasdegib or placebo plus cytarabine and daunorubicin in the intensive study, or with glasdegib or placebo plus azacitidine in the non-intensive study.

	Intensive study			Non-intensive study		
	Glasdegib + cytarabine + daunorubicin (*n* = 201)	Placebo + cytarabine + daunorubicin (*n* = 203)	Total (*N* = 404)	Glasdegib + azacitidine (*n* = 163)	Placebo + azacitidine (*n* = 162)	Total (*N* = 325)
Age, years						
Median (range)	59 (19–78)	59 (19–86)	59 (19–86)	73 (47–90)	73 (56–94)	73 (47–94)
≥65, *n* (%)	57 (28.4)	62 (30.5)	119 (29.5)	147 (90.2)	145 (89.5)	292 (89.8)
Sex, *n* (%)						
Male	130 (64.7)	106 (52.2)	236 (58.4)	97 (59.5)	89 (54.9)	186 (57.2)
Female	71 (35.3)	97 (47.8)	168 (41.6)	66 (40.5)	73 (45.1)	139 (42.8)
Race						
White	110 (54.7)	123 (60.6)	233 (57.7)	97 (59.5)	99 (61.1)	196 (60.3)
Black or African American	3 (1.5)	3 (1.5)	6 (1.5)	1 (0.6)	7 (4.3)	8 (2.5)
Asian	66 (32.8)	57 (28.1)	123 (30.4)	51 (31.3)	44 (27.2)	95 (29.2)
American Indian or Alaska native	1 (0.5)	0	1 (0.2)	0	0	0
Multiracial	1 (0.5)	0	1 (0.2)	0	0	0
Not reported	20 (10.0)	20 (9.9)	40 (9.9)	14 (8.6)	12 (7.4)	26 (8.0)
ECOG PS, *n* (%)						
0	87 (43.3)	85 (41.9)	172 (42.6)	43 (26.4)	38 (23.5)	81 (24.9)
1	93 (46.3)	98 (48.3)	191 (47.3)	80 (49.1)	78 (48.1)	158 (48.6)
2	15 (7.5)	16 (7.9)	31 (7.7)	36 (22.1)	37 (22.8)	73 (22.5)
3	4 (2.0)	1 (0.5)	5 (1.2)	3 (1.8)	8 (4.9)	11 (3.4)
4	0	0	0	1 (0.6)	1 (0.6)	2 (0.6)
Not reported	2 (1.0)	3 (1.5)	5 (1.2)	0	0	0
Primary AML diagnosis, *n*	199	203	402	163	162	325
De novo	158 (78.6)	169 (83.3)	327 (80.9)	118 (72.4)	120 (74.1)	238 (73.2)
Secondary	41 (20.4)	34 (16.7)	75 (18.6)	45 (27.6)	42 (25.9)	87 (26.8)
Time since onset, median (range), months	0.23 (0.03–27.14)	0.23 (0.03–93.67)	0.23 (0.03–93.67)	0.46 (0.03–2.33)	0.49 (0.03–37.85)	0.46 (0.03–37.85)
ELN risk group, *n* (%)						
Favorable	15 (7.5)	17 (8.4)	32 (7.9)	8 (4.9)	7 (4.3)	15 (4.6)
Intermediate	161 (80.1)	161 (79.3)	322 (79.7)	123 (75.5)	124 (76.5)	247 (76.0)
Adverse	25 (12.4)	25 (12.3)	50 (12.4)	32 (19.6)	31 (19.1)	63 (19.4)

*AML* acute myeloid leukemia, *ECOG PS* Eastern Cooperative Oncology Group performance status, *ELN* European LeukemiaNet.

In the intensive study, median (range) duration of study treatment was 10.4 (0.1–86.3) weeks in the glasdegib arm versus 10.3 (0.1–95.4) weeks in the placebo arm; mean (standard deviation [SD]) relative dose intensity was 85.0% (20.0%) for glasdegib versus 86.7% (19.4%) for placebo. In the non-intensive study, median (range) duration of study treatment was 22.2 (0.4–156.6) weeks in the glasdegib arm versus 24.2 (0.4–127.3) weeks in the placebo arm; mean (SD) relative dose intensity was 87.1% (16.4%) for glasdegib versus 87.2% (17.4%) for placebo. A total of 126 (31.6%) patients in the intensive study and 163 (50.6%) in the non-intensive study received study treatment for ≥24 weeks.

### Efficacy

#### Intensive study

Similar percentages of patients in the glasdegib versus placebo arms achieved CR (49.3% [*n* = 99/201] vs. 47.3% [*n* = 96/203]), CRi (1.5% [*n* = 3/201] vs. 5.4% [*n* = 11/203]), CR_MRD-neg_ (5.0% [*n* = 10/201] vs. 5.4% [*n* = 11/203]), or PR (5.0% [*n* = 10/201] vs. 4.4% [*n* = 9/203]), or had progressive disease (7.5% [*n* = 15/201] vs. 7.4% [*n* = 15/203]) (Table 2).

**Table 2:** Best overall response to treatment with glasdegib or placebo plus cytarabine and daunorubicin in the intensive study, and to glasdegib or placebo plus azacitidine in the non-intensive study.

	Intensive study (*N* = 404)				Non-intensive study (*N* = 325)			
	Glasdegib + cytarabine + daunorubicin (*n* = 201)		Placebo + cytarabine + daunorubicin (*n* = 203)		Glasdegib + azacytidine (*n* = 163)		Placebo + azacytidine (*n* = 162)	
	*n* (%)	95% Exact CI	*n* (%)	95% Exact CI	*n* (%)	95% Exact CI	*n* (%)	95% Exact CI
Best overall response								
CR_MRD-neg_	10 (5.0)	2.4–9.0	11 (5.4)	2.7–9.5	3 (1.8)	0.4–5.3	1 (0.6)	0.0–3.4
CR^a^	99 (49.3)	42.1–56.4	96 (47.3)	40.3–54.4	32 (19.6)	13.8–26.6	21 (13.0)	8.2–19.1
CRh^b^	NA	NA	NA	NA	5 (3.1)	1.0–7.0	5 (3.1)	1.0–7.1
CRi^c^	3 (1.5)	0.3–4.3	11 (5.4)	2.7–9.5	4 (2.5)	0.7–6.2	1 (0.6)	0.0–3.4
PR	10 (5.0)	2.4–9.0	9 (4.4)	2.0–8.2	4 (2.5)	0.7–6.2	8 (4.9)	2.2–9.5
MFLS	3 (1.5)	0.3–4.3	4 (2.0)	0.5–5.0	5 (3.1)	1.0–7.0	1 (0.6)	0.0–3.4
Stable disease	24 (11.9)	7.8–17.2	22 (10.8)	6.9–15.9	30 (18.4)	12.8–25.2	41 (25.3)	18.8–32.7
Indeterminate response	5 (2.5)	0.8–5.7	5 (2.5)	0.8–5.7	15 (9.2)	5.2–14.7	11 (6.8)	3.4–11.8
Progressive disease	15 (7.5)	4.2–12.0	15 (7.4)	4.2–11.9	9 (5.5)	2.6–10.2	12 (7.4)	3.9–12.6
Not evaluable	32 (15.9)	-	30 (14.8)	-	56 (34.4)	-	61 (37.7)	-
Stratified analysis of response (glasdegib vs. placebo arms)^d^								
CR_MRD-neg_ + CR + CRh	NA	NA	NA	NA	40 (24.5)	18.1–31.9	27 (16.7)	11.3–23.3
Odds ratio (95% CI); *p*-value^e^	NA				1.624 (0.941–2.804); *p* = 0.081			
CR_MRD-neg_ + CR + CRh + CRi	NA	NA	NA	NA	44 (27.0)	20.3–34.5	28 (17.3)	11.8–24.0
Odds ratio (95% CI); *p*-value^e^	NA				1.767 (1.037–3.013); *p* = 0.036			
CR_MRD-neg_ + CR	109 (54.2)	47.1–61.3	107 (52.7)	45.6–59.7	NA	NA	NA	NA
Odds ratio (95% CI); *p*-value^e^	1.068 (0.711–1.605); *p* = 0.754				NA			
CR_MRD-neg_ + CR + CRi	112 (55.7)	48.6–62.7	118 (58.1)	51.0–65.0	NA	NA	NA	NA
Odds ratio (95% CI); *p*-value^e^	0.897 (0.597–1.349); *p* = 0.605				NA			

*p*-values are two-sided.

*CI* confidence interval, *CR* complete remission, *CRh* complete remission with partial hematologic recovery, *CRi* complete remission with incomplete hematologic recovery, *CR*_MRD-neg_, complete remission without measurable residual disease, *ELN* European LeukemiaNet, *MLFS* morphologic leukemia-free state, *MRD* measurable residual disease, *PR* partial remission.

^a^CR included patients who were MRD positive or unknown.

^b^CRh criteria: MRD positive or unknown; both neutrophil ≥0.5 × 109/L and platelets ≥50 × 109/L but does not satisfy both neutrophils ≥1 × 109/L and platelets ≥100 × 109/L at the same time; absence of extramedullary disease; and absence of blasts with Auer rods.

^c^CRi criteria: MRD positive or unknown; either neutropenia ( <1 × 109/L) or platelets <100 × 109/L must be met for the intensive study, or neutrophils <0.5 × 109/L or platelets <50 × 109/L for the non-intensive study; absence of extramedullary disease; and absence of blasts with Auer rods.

^d^Data stratified by age group ( <60 vs. ≥60 years), ELN risk category, and region (rest-of-world vs. China) for the intensive study, and by age group ( <75 vs. ≥75 years) and ELN risk category for the non-intensive study. This analysis adjusts for the confounding factors of age, ELN risk category, and region (for the intensive study) when calculating the odds ratio for achieving the specified response.

^e^Statistics are from Cochran-Mantel-Haenszel stratified test.

**Fig. 3:**
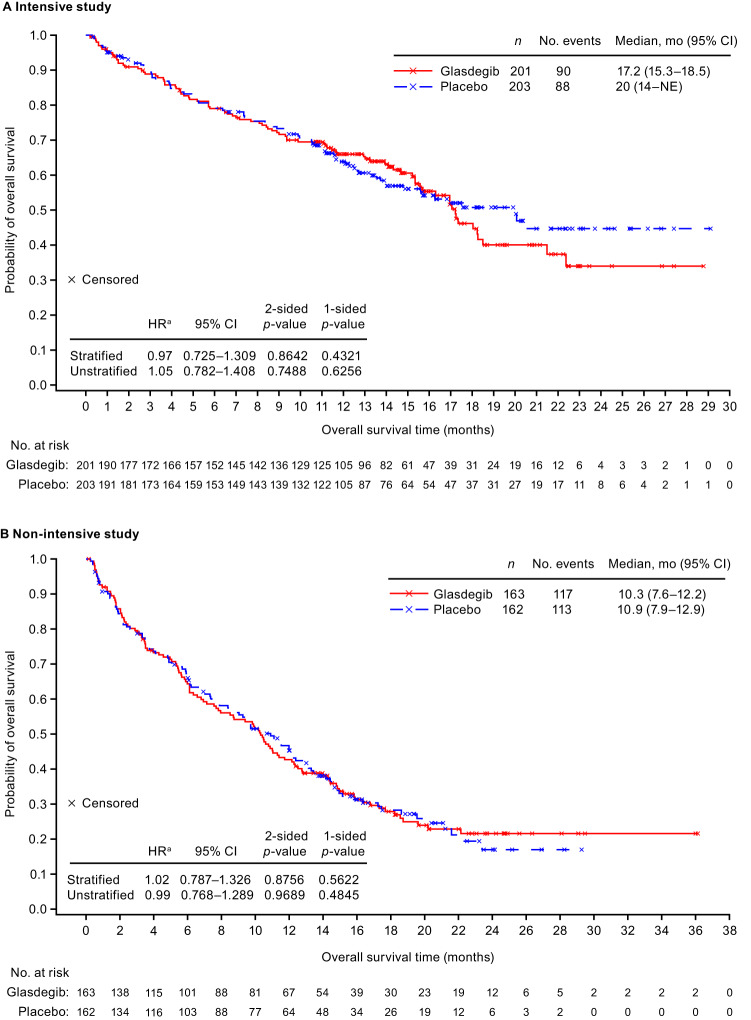
Overall survival in the intensive and non-intensive studies. **A** Overall survival in patients treated with glasdegib or placebo plus cytarabine and daunorubicin. **B** Overall survival in patients treated with glasdegib or placebo plus azacitidine. ^a^Glasdegib vs. placebo. CI confidence interval, HR hazard ratio, OS overall survival.

Median (range) follow-up for OS, which included all patients, was 12.7 (0.2–28.8) months in the glasdegib arm and 12.2 (0.3–29.1) months in the placebo arm. OS (unstratified) was similar between arms (HR 1.05 [95% CI: 0.782–1.408]; two-sided *p* = 0.749) (Fig. 3A), but favored placebo for patients with intermediate ELN risk (HR 1.78 [95% CI: 1.041–3.045]; two-sided *p* = 0.033) and Asian patients (HR 2.74 [95% CI: 1.365–5.509]; two-sided *p* = 0.003) (Fig. S1).

#### Non-intensive study

CR occurred in 19.6% (*n* = 32/163) of patients in the glasdegib arm and 13.0% (*n* = 21/162) in the placebo arm (Table 2). Few patients in the glasdegib versus placebo arm achieved CR_MRD-neg_ (1.8% [*n* = 3/163] vs. 0.6% [*n* = 1/162]), CRi (2.5% [*n* = 4/163] vs. 0.6% [*n* = 1/162]), or CRh (3.1% [*n* = 5/163] vs. 3.1% [*n* = 5/162]). The incidence of progressive disease was similar between arms (5.5% [*n* = 9/163] vs. 7.4% [*n* = 12/162]).

The probability of achieving CR_MRD-neg_, CR, CRh, or CRi favored glasdegib when stratified by age and ELN risk (odds ratio 1.767 [95% CI: 1.037–3.013]; two-sided *p* = 0.036; Table 2). In the glasdegib versus placebo arms, median (range) time to achieve CRi or better was 3.8 (0.9–10.4) versus 3.8 (0.9–11.3) months, and median (range) time to achieve CRh or better was 3.9 (1.9–10.4) versus 3.8 (0.9–11.3) months; 22.1% (*n* = 36/163) versus 16.0% (*n* = 26/162) achieved CRi or better, and 19.0% (*n* = 31/163) versus 15.4% (*n* = 25/162) achieved CRh or better, within six months of treatment.

Median (range) follow-up for OS, which included all patients, was 10.0 (0.2–36.1) months in the glasdegib arm and 9.7 (0.3–29.3) months in the placebo arm. OS (unstratified) was similar in both arms (HR 0.99 [95% CI 0.768–1.289]; two-sided *p* = 0.969) (Fig. 3B). OS did not differ between treatment groups when compared with each other based on various baseline characteristics (two-sided *p* > 0.05; Fig. S2).

### Safety and tolerability

The rate of treatment-emergent AEs (TEAEs) was similar between treatment arms in both studies (Table S2). The most common TEAEs (glasdegib vs. placebo) were nausea, febrile neutropenia, and anemia in the intensive study, and anemia, constipation, and nausea in the non-intensive study (Tables 3, 4). Across both studies, there was considerable variability in platelet and neutrophil counts within each treatment arm, but the median values were generally similar between arms across time **(**Figs. S3, S4).

**Table 3:** All-causality, treatment-emergent adverse events occurring in ≥20% of patients in either treatment arm in the intensive study.

*n* (%)	Glasdegib + cytarabine + daunorubicin (*n* = 198)		Placebo + cytarabine + daunorubicin (*n* = 201)		Total (*N* = 399)	
	Any grade	Grade 3/4	Any grade	Grade 3/4	Any grade	Grade 3/4
Any adverse event	196 (99.0)	173 (87.4)	198 (98.5)	169 (84.1)	394 (98.7)	342 (85.7)
Nausea	110 (55.6)	4 (2.0)	108 (53.7)	4 (2.0)	218 (54.6)	8 (2.0)
Febrile neutropenia	106 (53.5)	106 (53.5)	107 (53.2)	107 (53.2)	213 (53.4)	213 (53.4)
Anemia	106 (53.5)	92 (46.5)	101 (50.2)	86 (42.8)	207 (51.9)	178 (44.6)
Diarrhea	98 (49.5)	7 (3.5)	88 (43.8)	6 (3.0)	186 (46.6)	13 (3.3)
Pyrexia	83 (41.9)	8 (4.0)	87 (43.3)	12 (6.0)	170 (42.6)	20 (5.0)
Hypokalemia	76 (38.4)	22 (11.1)	84 (41.8)	26 (12.9)	160 (40.1)	48 (12.0)
Platelet count decreased	80 (40.4)	78 (39.4)	76 (37.8)	73 (36.3)	156 (39.1)	151 (37.8)
Constipation	71 (35.9)	2 (1.0)	61 (30.3)	1 (0.5)	132 (33.1)	3 (0.8)
White blood cell count decreased	65 (32.8)	63 (31.8)	54 (26.9)	51 (25.4)	119 (29.8)	114 (28.6)
Neutrophil count decreased	57 (28.8)	56 (28.3)	52 (25.9)	51 (25.4)	109 (27.3)	107 (26.8)
Thrombocytopenia	52 (26.3)	52 (26.3)	54 (26.9)	52 (25.9)	106 (26.6)	104 (26.1)
Vomiting	58 (29.3)	1 (0.5)	41 (20.4)	1 (0.5)	99 (24.8)	2 (0.5)
Rash	46 (23.2)	2 (1.0)	50 (24.9)	2 (1.0)	96 (24.1)	4 (1.0)
Decreased appetite	52 (26.3)	2 (1.0)	42 (20.9)	9 (4.5)	94 (23.6)	11 (2.8)
ALT increased	39 (19.7)	17 (8.6)	54 (26.9)	12 (6.0)	93 (23.3)	29 (7.3)
Headache	40 (20.2)	0	49 (24.4)	4 (2.0)	89 (22.3)	4 (1.0)
Hypophosphatemia	43 (21.7)	27 (13.6)	44 (21.9)	23 (11.4)	87 (21.8)	50 (12.5)
Neutropenia	41 (20.7)	39 (19.7)	46 (22.9)	43 (21.4)	87 (21.8)	82 (20.6)
Pneumonia	42 (21.2)	34 (17.2)	41 (20.4)	34 (16.9)	83 (20.8)	68 (17.0)
AST increased	31 (15.7)	12 (6.1)	42 (20.9)	9 (4.5)	73 (18.3)	21 (5.3)
Stomatitis	29 (14.6)	3 (1.5)	41 (20.4)	5 (2.5)	70 (17.5)	8 (2.0)

*ALT* alanine aminotransferase, *AST* aspartate aminotransferase.

**Table 4:** All-causality, treatment-emergent adverse events occurring in ≥20% of patients in either treatment arm in the non-intensive study.

*n* (%)	Glasdegib + cytarabine + daunorubicin (*n* = 162)		Placebo + cytarabine + daunorubicin (*n* = 160)		Total (*n* = 322)	
	Any grade	Grade 3/4	Any grade	Grade 3/4	Any grade	Grade 3/4
Any adverse event	161 (99.4)	106 (65.4)	158 (98.8)	100 (62.5)	319 (99.1)	206 (64.0)
Anemia	75 (46.3)	64 (39.5)	73 (45.6)	60 (37.5)	148 (46.0)	124 (38.5)
Constipation	59 (36.4)	2 (1.2)	52 (32.5)	2 (1.3)	111 (34.5)	4 (1.2)
Nausea	58 (35.8)	2 (1.2)	44 (27.5)	1 (0.6)	102 (31.7)	3 (0.9)
Pneumonia	43 (26.5)	27 (16.7)	48 (30.0)	38 (23.8)	91 (28.3)	65 (20.2)
Pyrexia	48 (29.6)	9 (5.6)	42 (26.3)	6 (3.8)	90 (28.0)	15 (4.7)
Febrile neutropenia	42 (25.9)	42 (25.9)	40 (25.0)	39 (24.4)	82 (25.5)	81 (25.2)
Thrombocytopenia	39 (24.1)	34 (21.0)	35 (21.9)	32 (20.0)	74 (23.0)	66 (20.5)
Diarrhea	40 (24.7)	5 (3.1)	33 (20.6)	4 (2.5)	73 (22.7)	9 (2.8)
Neutropenia	38 (23.5)	35 (21.6)	30 (18.8)	27 (16.9)	68 (21.1)	62 (19.3)
Vomiting	36 (22.2)	1 (0.6)	32 (20.0)	0	68 (21.1)	1 (0.3)
Decreased appetite	45 (27.8)	5 (3.1)	21 (13.1)	2 (1.3)	66 (20.5)	7 (2.2)
Hypokalemia	35 (21.6)	19 (11.7)	22 (13.8)	10 (6.3)	57 (17.7)	29 (9.0)
Weight decreased	37 (22.8)	14 (8.6)	19 (11.9)	5 (3.1)	56 (17.4)	19 (5.9)
Dysgeusia	38 (23.5)	0	8 (5.0)	0	46 (14.3)	0

Grade 3/4 TEAEs and serious TEAEs occurred with similar frequency in glasdegib- versus placebo-treated patients in both studies (Tables 3, 4, S2). The most common serious TEAEs in the glasdegib versus placebo arms were febrile neutropenia (9.1% [*n* = 18/198] vs. 8.5% [*n* = 17/201]), sepsis (7.6% [*n* = 15/198] vs. 6.5% [*n* = 13/201]), and pneumonia (7.6% [*n* = 15/198] vs. 5.5% [*n* = 11/201]) in the intensive study, and pneumonia (17.9% [*n* = 29/162] vs. 22.5% [*n* = 36/160]), febrile neutropenia (14.8% [*n* = 24/162] vs. 12.5% [*n* = 20/160]), and sepsis (8.6% [*n* = 14/162] vs. 6.3% [*n* = 10/160]) in the non-intensive study. The rates of permanent discontinuation due to TEAEs and dose modifications were similar with glasdegib versus placebo in both studies (Table S2).

A total of 178 (44.1%) patients in the intensive study and 230 (70.8%) in the non-intensive study died. Deaths were balanced between glasdegib and placebo arms (intensive: 44.8% [*n* = 90/201] vs. 43.3% [*n* = 88/203]; non-intensive: 71.8% [*n* = 117/163] vs. 69.8% [*n* = 113/162]). In both studies, disease progression was the most common cause of death for both glasdegib and placebo (intensive: 25.9% [*n* = 52/201] vs. 24.1% [*n* = 49/203]; non-intensive: 42.3% [*n* = 69/163] vs. 42.0% [*n* = 68/162]). Treatment toxicity caused seven deaths (1.7%; *n* = 4 glasdegib, *n* = 3 placebo) in the intensive study and four deaths (1.2%; *n* = 2 in each arm) in the non-intensive study.

Fatigue was reported as a TEAE in 64 (16.0%) patients in the intensive study and 39 (12.1%) patients in the non-intensive study. In both studies, the incidence of fatigue was similar between glasdegib versus placebo: 15.7% versus 16.4% in the intensive study; 9.3% versus 15.0% in the non-intensive study. Over 80% of patients in both arms reported no improvement in fatigue after 8 weeks in the intensive study (glasdegib vs placebo, 82.6% [*n* = 166/201] vs 82.8% [*n* = 168/203]) and after 12 weeks in the non-intensive study (glasdegib vs placebo, 88.3% [*n* = 144/163] vs 84.6% [*n* = 137/162]).

### Pharmacokinetics

In the intensive study, geometric mean (%coefficient of variation) C_trough_ values for glasdegib were 413.54 (125) ng/mL on induction Day 10 (*n* = 81), 245.48 (80) ng/mL on first-consolidation Day 1 (*n* = 33), and 259.79 (122) ng/mL on second-consolidation Day 1 (*n* = 41). In the non-intensive study, corresponding values for glasdegib were 565.44 (126) ng/mL on Cycle 1 Day 15 (*n* = 34) and 472.42 (122) ng/mL on Cycle 2 Day 1 (*n* = 37). All patients were receiving continuous glasdegib at the time of pharmacokinetic sampling.

### Biomarkers

In an analysis examining the association of genetic abnormalities defined in the 2017 ELN stratification criteria [10] with improved OS in the glasdegib or placebo arm, none of the ELN risk groups (assayed locally) had a significantly better outcome with glasdegib compared with placebo in the intensive and non-intensive studies (Fig. S5**;** Fig. S6). However, in the intensive study, patients treated with placebo in the intermediate risk group had a better outcome than patients treated with glasdegib (two-sided *p* = 0.010; Fig. S5). Similarly, in the non-intensive study, those in the adverse risk group (two-sided *p* = 0.009) and specifically those with mutated *RUNX1* (two-sided *p* = 0.014) had a better outcome when treated with placebo than glasdegib (Fig. S6).

**Fig. 4:**
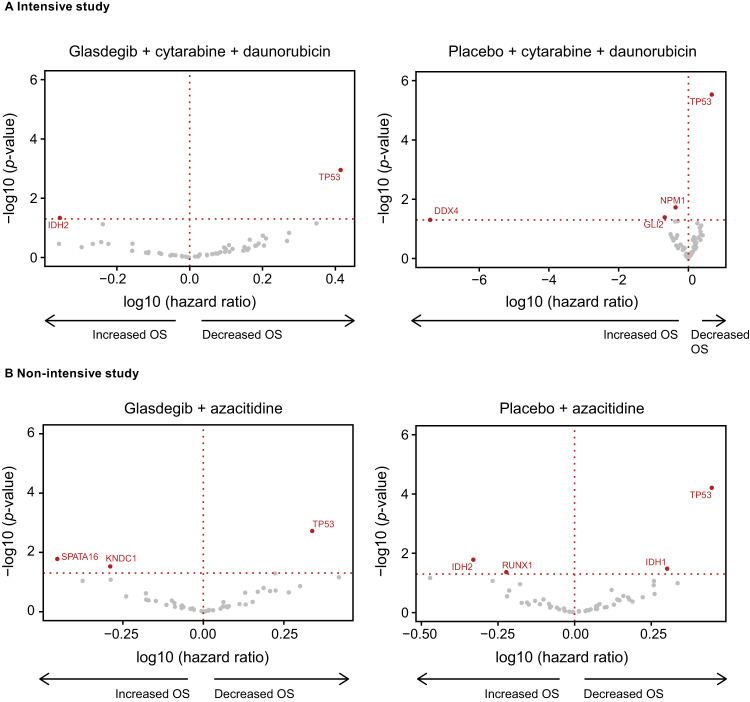
Volcano plots showing the association between gene mutation and overall survival. **A** Intensive and **B** non-intensive studies. OS overall survival.

Whole exome-sequencing (WES) was performed centrally with bone marrow aspirate samples collected at screening for 308 and 267 patients in the intensive and non-intensive studies, respectively. Mutations in the ELN risk stratification [10] that could be analyzed in the WES analysis included *NPM1*, *FLT3*, *CEBPA*, *RUNX1*, *ASXL1*, and *TP53*. None of the mutations examined were associated with improved OS in the glasdegib arm in either study (Fig. 4).

An interaction analysis was performed for each study to determine whether any mutations showed a differential association with OS between the glasdegib and placebo arms. The only gene with a significant interaction *p*-value in the intensive study was *GLI2*, wherein mutations were associated with increased OS in the placebo but not glasdegib arms (*n* = 30/307 [9.8%], *p*_interaction_ = 0.013) (Fig. S7A). The distribution of mutations within the *GLI2* gene in the intensive study is shown in Fig. S8. In the non-intensive study, patients with the *RUNX1* mutation (*n* = 53/267 [19.9%]) had increased OS in the placebo arm but not in the glasdegib arm (*p*_interaction_ = 0.0005; Fig. S7B), and those with the *IDH1* mutation (*n* = 27/267 [10.1%]) had decreased OS in the placebo arm but not in the glasdegib arm (*p*_interaction_ = 0.018; Fig. S7C).

## Discussion

This randomized phase 3 trial in patients with untreated AML found that the addition of glasdegib versus placebo to cytarabine and daunorubicin or azacitidine therapy did not significantly improve OS, and the primary efficacy endpoint for the BRIGHT AML 1019 phase 3 trial was not met. The results of this trial emphasize the importance of phase 3 confirmatory studies, and of conducting those studies in heterogenous populations.

In the non-intensive study, adding glasdegib to azacitidine resulted in a numerically higher rate of CR compared with azacitidine alone, and the probability of achieving CR_MRD-neg_, CR, CRh, or CRi was significantly greater for the glasdegib versus placebo treatment arm. Similarly, the phase 2 BRIGHT AML 1003 trial found that the CR rate increased when adding glasdegib to LDAC versus LDAC alone (17.0% vs. 2.3%) in newly diagnosed patients who were ineligible for intensive chemotherapy [23]. The difference in the CR rates between the glasdegib and placebo treatment groups was smaller in the current study, largely due to the higher rate observed with azacitidine alone in this trial (13.0%) versus LDAC alone in the phase 2 trial (2.3%). In the intensive study, the CR rate was similar between the glasdegib and placebo arms, and there were no significant differences between treatment groups in the probability of achieving CR_MRD-neg_/CR or CR_MRD-neg_/CR/CRi.

The phase 2 BRIGHT AML 1003 trial also revealed a survival benefit with glasdegib added to LDAC versus LDAC alone [23] that was not observed in the current trial when glasdegib was combined with cytarabine and daunorubicin or azacitidine therapy. The possible reasons for differences in the efficacy of glasdegib between the phase 2 trial and the current trial are manifold. First, the size of the patient population increased from 132 to 729, which may have affected other factors that contribute to outcomes such as patient selection and appropriate supportive care. Secondly, the chemotherapy partner to glasdegib changed from LDAC in the phase 2 trial to either intensive 7 + 3 therapy or azacitidine in the current trial, and this change may have compromised efficacy. The mechanism for better efficacy when combined with LDAC could reflect a possible synergy with cytarabine alone (specifically at low doses), lower efficacy of LDAC (which leaves more room for improvement with the addition of glasdegib), or other factors. Additionally, the efficacy of glasdegib in the current setting could be a matter of finding the correct treatment schedule or sequence of the chemotherapy approaches used here. Lastly, in contrast to the phase 2 trial, the current trial was placebo-controlled, which could impact therapy duration and consequent efficacy.

The overall safety profile of glasdegib plus cytarabine and daunorubicin or azacitidine was consistent with the known safety profiles of these agents. The overall rate of TEAEs, grade 3/4 TEAEs, and serious TEAEs was generally similar between treatment arms in the intensive and non-intensive studies.

In the intensive study, *GLI2* mutations were associated with improved survival in the placebo arm but not the glasdegib arm. *GLI2* is a downstream transcription factor of Smoothened, and a key mediator of Hedgehog signaling [28]. As these *GLI2* mutations were distributed throughout the gene, they may interfere with its activities, including DNA binding, transcriptional activation, and binding to its target Sufu. We speculate these mutations may decrease its transcriptional activity or modulate localization to the cilia. However, as *GLI2* activity can also be modulated by other signaling pathways (e.g., TBF-β signaling) [29], inhibition of hedgehog signaling may increase activity of other compensatory signaling pathways (e.g., TBF-β), which may sufficiently activate *GLI2* and potentially reduce the effects of Smoothened inhibition.

In conclusion, this study found the addition of glasdegib to either cytarabine and daunorubicin or azacitidine therapy did not significantly improve OS, and the primary endpoint for the BRIGHT AML 1019 phase 3 trial was not met. These results are in contrast with favorable results previously observed with glasdegib in combination with LDAC compared with LDAC alone [23]. The current trial, however, confirmed the acceptable safety profile of glasdegib and did not identify any new safety signals.

### Supplementary information

Supplemental material

## Data Availability

Upon request, and subject to review, Pfizer will provide the data that support the findings of this study. Subject to certain criteria, conditions and exceptions, Pfizer may also provide access to the related individual de-identified participant data. See https://www.pfizer.com/science/clinical-trials/trial-data-and-results for more information.
