# Supplementary material for: Glasdegib plus intensive or non-intensive chemotherapy for untreated acute myeloid leukemia: results from the randomized, phase 3 BRIGHT AML 1019 trial
Source: Leukemia. 2023 Aug 21;37(10):2017–26. doi: 10.1038/s41375-023-02001-z (PMC10539167; doi:10.1038/s41375-023-02001-z)

SUPPLEMENTARY INFORMATION

Assessments and Analyses

Safety assessments included adverse events (AEs) graded according to the Common Terminology Criteria for AEs (CTCAE) v4.03.

Modified European LeukemiaNet (ELN) [1] response assessments included evaluation of bone marrow, peripheral blood neutrophils, platelets, and percent blasts, and assessment for extramedullary disease. Measurable residual disease status was assessed centrally at each bone marrow assessment by multiparametric flow cytometry. Every patient underwent an Investigator-determined response assessment. A response was marked as indeterminate if the evaluation did not meet specific ELN response criteria for complete remission (CR), CR with incomplete hematologic recovery, morphologic leukemia-free state, partial remission, stable disease (SD), or progressive disease, or CR with partial hematologic recovery as previously defined [2]; for example, when SD was not maintained for 3 months, a bone marrow assessment was missing, or if all requirements were unmet.

For pharmacokinetic analyses, a sparse pharmacokinetic sampling approach was employed, with pharmacokinetic samples collected during induction/consolidation (intensive study) and during Cycles 1–3 (non-intensive study).

Bone marrow aspirate samples were collected at screening, frozen, and sequenced (Illumina NovaSeq). Biomarker analyses focused on 115 genes with a known role in acute myeloid leukemia/myelodysplastic syndrome or the Hedgehog signaling pathway (the same genes analyzed for the phase 1b study [3]). The resulting DNA sequences from whole exome sequencing data were processed by the Personalis ACE (Accuracy and Content Enhanced) Cancer Exome pipeline (Personalis Inc, Menlo Park, CA), which uses Burrows Wheeler Aligner (BWA), Genome Analysis Toolkit (GATK), Mutect, VarDict, and Picard for variant calling. Mutations with at least five mutant reads that were not annotated as synonymous variants and that were annotated as resulting in a change in protein coding sequence were included in the analysis.

An additional analysis was performed with each of the genetic abnormalities defined in the 2017 ELN stratification criteria [1] to determine if they were associated with improved overall survival in the glasdegib or placebo arm. This analysis was only performed for genetic abnormalities that were reported with an occurrence of at least five.

Relative dose intensity (%) was calculated as 100 * [dose intensity (mg/day)] / [100 (mg/day)], where dose intensity is [cumulative dose (mg)] / [treatment duration (days), including any dose interruption period prior to permanent treatment discontinuation and excluding the hematopoietic stem cell transplantation period, if applicable].

End of trial

The phase 3 BRIGHT AML 1019 trial did not end prematurely. However, investigators opted to remove patients from the study once the primary objective in each cohort was not met while remaining patients continued to be followed up for safety. Some secondary and exploratory endpoints could not be analyzed as originally planned once it became apparent that the trial did not meet its primary objective.

References

[1] Dohner H, Estey E, Grimwade D, Amadori S, Appelbaum FR, Buchner T, et al. Diagnosis and management of AML in adults: 2017 ELN recommendations from an international expert panel. Blood. 2017; 129:424-447. https://doi.org/10.1182/blood-2016-08-733196.

[2] Kantarjian H, Stein A, Gökbuget N, Fielding AK, Schuh AC, Ribera J-M, et al. Blinatumomab versus chemotherapy for advanced acute lymphoblastic leukemia. N Engl J Med. 2017; 376:836-847. https://doi.org/0.1056/NEJMoa1609783.

[3] Sekeres MA, Schuster M, Joris M, Krauter J, Maertens J, Breems D, et al. A phase 1b study of glasdegib + azacitidine in patients with untreated acute myeloid leukemia and higher-risk myelodysplastic syndromes. Ann Hematol. 2022; 101:1689-1701. https://doi.org/10.1007/s00277-022-04853-4.

Supplementary Table S1 Baseline genetic abnormalities in the intensive and non-intensive studies

	Intensive study		Non-intensive study	
**Genetic abnormality**	**Glasdegib + cytarabine / daunorubicin (*n* = 201)**	**Placebo + cytarabine / daunorubicin (*n* = 203)**	**Glasdegib + azacitidine (*n* = 163)**	**Placebo + azacitidine (*n* = 162)**
Cytogenetic abnormalities not classified as favorable or adverse	68 (33.8)	62 (30.5)	70 (42.9)	75 (46.3)
Complex karyotype	37 (18.4)	31 (15.3)	27 (16.6)	31 (19.1)
-7	28 (13.9)	26 (12.8)	21 (12.9)	21 (13.0)
-5 or del(5q)	24 (11.9)	18 (8.9)	23 (14.1)	24 (14.8)
NPM1 mutation without *FLT3*-ITD	20 (10.0)	26 (12.8)	13 (8.0)	11 (6.8)
t(8;21)(q22;q22.1); *RUNX1-RUNX1T1*	12 (6.0)	15 (7.4)	3 (1.8)	2 (1.2)
Wild-type *NPM1* without *FLT3*-ITD (without adverse-risk genetic lesions)	12 (6.0)	14 (6.9)	15 (9.2)	6 (3.7)
Mutated *TP53*	12 (6.0)	14 (6.9)	7 (4.3)	9 (5.6)
-17/abn(17p)	12 (6.0)	11 (5.4)	10 (6.1)	11 (6.8)
Monosomal karyotype	10 (5.0)	12 (5.9)	8 (4.9)	9 (5.6)
Mutated *RUNX1*	9 (4.5)	14 (6.9)	9 (5.5)	11 (6.8)
Mutated *ASXL1*	6 (3.0)	15 (7.4)	3 (1.8)	5 (3.1)
NPM1 mutation with *FLT3*-ITD^{low}	6 (3.0)	5 (2.5)	4 (2.5)	2 (1.2)
Mutated *NPM1* and *FLT3*-ITD^{high}	6 (3.0)	3 (1.5)	4 (2.5)	1 (0.6)
Biallelic mutated *CEBPA*	5 (2.5)	12 (5.9)	4 (2.5)	4 (2.5)
inv(16)(p13.1q22); *CBFB-MYH11*	5 (2.5)	6 (3.0)	5 (3.1)	2 (1.2)
t(v;11q23.3); *KMT2A* rearranged	5 (2.5)	4 (2.0)	2 (1.2)	2 (1.2)
Wild-type *NPM1* and *FLT3*-ITD^{high}	3 (1.5)	5 (2.5)	4 (2.5)	0
t(9;11)(p21.3;q23.3); *MLLT3-KMT2A*	2 (1.0)	4 (2.0)	1 (0.6)	2 (1.2)
inv(3)(q21.3q26.2); *GATA2,MECOM*(*EVI1*)	1 (0.5)	2 (1.0)	1 (0.6)	0
t(6;9)(p23;q34.1); *DEK-NUP214*	1 (0.5)	2 (1.0)	1 (0.6)	0
Wild-type *NPM1* with *FLT3*-ITD^{low} (without adverse-risk genetic lesions)	1 (0.5)	1 (0.5)	4 (2.5)	10 (6.2)
t(3;3)(q21.3;q26.2); *GATA2,MECOM*(*EVI1*)	0	3 (1.5)	1 (0.6)	0
t(9;22)(q34.1;q11.2); *BCR-ABL1*	0	1 (0.5)	0	0

Supplementary Table S2. Treatment-emergent adverse event summary in patients treated with glasdegib or placebo plus cytarabine and daunorubicin in the intensive study, and with glasdegib or placebo plus azacitidine in the non-intensive study

	Intensive study			Non-intensive study		
n **(%)**	**Glasdegib + cytarabine + daunorubicin (*n* = 198)**	**Placebo + cytarabine + daunorubicin (*n* = 201)**	**Total (*N* = 399)**	**Glasdegib + azacitidine (*n* = 162)**	**Placebo + azacitidine (*n* = 160)**	**Total (*N* = 322)**
Patients with TEAEs	196 (99.0)	198 (98.5)	394 (98.7)	161 (99.4)	158 (98.8)	319 (99.1)
Patients with serious TEAEs	86 (43.4)	92 (45.8)	178 (44.6)	117 (72.2)	124 (77.5)	241 (74.8)
Patients with maximum grade 3/4 TEAEs	173 (87.4)	169 (84.1)	342 (85.7)	106 (65.4)	100 (62.5)	206 (64.0)
Patients with maximum grade 5 TEAEs	16 (8.1)	20 (10.0)	36 (9.0)	50 (30.9)	52 (32.5)	102 (31.7)
Patients with TEAEs leading to dose reduction of any study intervention	29 (14.6)	24 (11.9)	53 (13.3)	25 (15.4)	13 (8.1)	38 (11.8)
Patients with TEAEs leading to dose reduction of glasdegib/placebo	29 (14.6)	20 (10.0)	49 (12.3)	19 (11.7)	11 (6.9)	30 (9.3)
Patients with TEAEs leading to temporary discontinuation of any study intervention	62 (31.3)	56 (27.9)	118 (29.6)	104 (64.2)	102 (63.8)	206 (64.0)
Patients with TEAEs leading to temporary discontinuation of glasdegib/placebo	62 (31.3)	53 (26.4)	115 (28.8)	80 (49.4)	75 (46.9)	155 (48.1)
Patients with TEAEs leading to permanent discontinuation of any study intervention	26 (13.1)	29 (14.4)	55 (13.8)	67 (41.4)	63 (39.4)	130 (40.4)
Patients with TEAEs leading to permanent discontinuation of glasdegib/placebo	26 (13.1)	29 (14.4)	55 (13.8)	64 (39.5)	58 (36.3)	122 (37.9)

AE, adverse event; TEAE, treatment-emergent adverse event

Supplementary Fig. S1 Overall survival by baseline characteristics, in patients treated with glasdegib or placebo plus cytarabine and daunorubicin in the intensive study

Subgroup	*n* (%) Glasdegib + cytarabine + daunorubicin	*n* (%) Placebo + cytarabine + daunorubicin	No. events/*n* Glasdegib + cytarabine + daunorubicin	No. events/*n* Placebo + cytarabine + daunorubicin	Hazard ratio (95% CI)	1-sided *p*-value	2-sided *p*-value
All patients (stratified)	201 (100.0)	203 (100.0)	90/201	88/203	0.97 (0.725–1.309)	0.4321	0.8642
All patients (unstratified)	201 (100.0)	203 (100.0)	90/201	88/203	1.05 (0.782–1.408)	0.6256	0.7488
Derived ELN risk category:							
Favorable	46 (22.9)	54 (26.6)	9/46	19/54	0.62 (0.278–1.369)	0.1152	0.2304
Intermediate	79 (39.3)	70 (34.5)	37/79	21/70	1.78 (1.041–3.045)	0.9836	0.0327
Adverse	69 (34.3)	78 (38.4)	41/69	47/78	0.91 (0.598–1.388)	0.3324	0.6647
Age:							
≤60	111 (55.2)	114 (56.2)	35/111	35/114	1.05 (0.659–1.683)	0.5853	0.8295
>60	90 (44.8)	89 (43.8)	55/90	53/89	1.02 (0.697–1.483)	0.5329	0.9342
Gender:							
Male	130 (64.7)	106 (52.2)	60/130	52/106	0.96 (0.663–1.395)	0.4176	0.8352
Female	71 (35.3)	97 (47.8)	30/71	36/97	1.11 (0.683–1.800)	0.6619	0.6763
Race:							
White	110 (54.7)	123 (60.6)	44/110	64/123	0.70 (0.475–1.023)	0.0317	0.0633
Asian	66 (32.8)	57 (28.1)	29/66	11/57	2.74 (1.365–5.509)	0.9984	0.0032
Not reported	20 (10.0)	20 (9.9)	15/20	11/20	1.55 (0.710–3.402)	0.8680	0.2640
Region:							
US	59 (29.4)	64 (31.5)	26/59	34/64	0.81 (0.484–1.344)	0.2027	0.4054
EU	69 (34.3)	74 (36.5)	34/69	39/74	0.87 (0.552–1.387)	0.2844	0.5688
China	24 (11.9)	16 (7.9)	10/24	0/16	NE (NE–NE)	0.9983	0.0035
ROW	49 (24.4)	49 (24.1)	20/49	15/49	1.42 (0.725–2.782)	0.8479	0.3042
ECOG performance status at baseline:							
0	87 (43.3)	85 (41.9)	35/87	32/85	1.13 (0.698–1.823)	0.6886	0.6229
1	93 (46.3)	98 (48.3)	45/93	46/98	1.05 (0.695–1.582)	0.5894	0.8213
≥2	19 (9.5)	17 (8.4)	9/19	9/17	0.82 (0.315–2.152)	0.3454	0.6908
BM blasts at baseline:							
<30%	45 (22.4)	47 (23.2)	23/45	24/47	0.91 (0.514–1.617)	0.3754	0.7508
≥30%	145 (72.1)	141 (69.5)	61/145	58/141	1.07 (0.743–1.526)	0.6330	0.7341
Hematologic disease history:							
de novo	158 (78.6)	169 (83.3)	56/158	65/169	0.92 (0.642–1.313)	0.3193	0.6385
Secondary	41 (20.4)	34 (16.7)	33/41	23/34	1.25 (0.729–2.133)	0.7912	0.4177
Baseline WBC category (10^9/L):							
<10	127 (63.2)	131 (64.5)	57/127	62/131	0.88 (0.614–1.261)	0.2432	0.4864
≥10	73 (36.3)	68 (33.5)	33/73	26/68	1.40 (0.837–2.352)	0.9007	0.1985

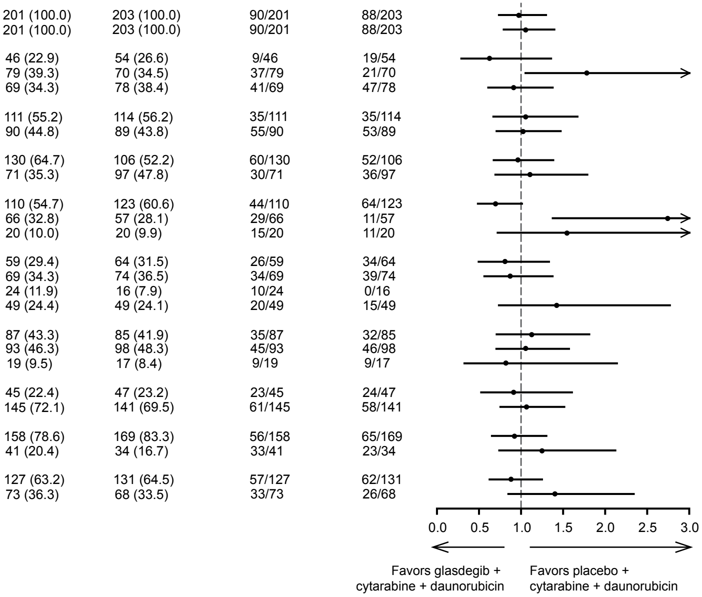

Percentages calculated based on the number of patients in the full analysis set in each treatment group.
Races (Black or African American, American Indian or Alaska Native, and multiracial) with too small number of patients (≤10) were removed from race categories.
BM, bone marrow; CI, confidence interval; ECOG, Eastern Cooperative Oncology Group; ELN, European LeukemiaNet; ROW, rest of world; WBC, white blood cell count

Supplementary Fig. S2 Overall survival by baseline characteristics, in patients treated with glasdegib or placebo plus azacitidine in the non-intensive study

Subgroup	*n* (%) Glasdegib + azacitidine	*n* (%) Placebo + azacitidine	No. of events/*n* Glasdegib + azacitidine	No. of events/*n* Placebo + azacitidine	Hazard ratio (95% CI)	1-sided *p*-value	2-sided *p*-value
All patients (stratified)	163 (100.0)	162 (100.0)	117/163	113/162	1.02 (0.787–1.326)	0.5622	0.8756
All patients (unstratified)	163 (100.0)	162 (100.0)	117/163	113/162	0.99 (0.768–1.289)	0.4845	0.9689
Derived ELN risk category:							
Favorable	28 (17.2)	19 (11.7)	16/28	12/19	0.90 (0.418–1.922)	0.3894	0.7789
Intermediate	76 (46.6)	77 (47.5)	50/76	50/77	0.90 (0.609–1.336)	0.3035	0.6070
Adverse	58 (35.6)	63 (38.9)	50/58	49/63	1.23 (0.830–1.830)	0.8502	0.2995
Age:							
<75	93 (57.1)	96 (59.3)	67/93	65/96	1.00 (0.710–1.405)	0.4971	0.9943
≥75	70 (42.9)	66 (40.7)	50/70	48/66	0.99 (0.668–1.475)	0.4851	0.9702
Gender:							
Male	97 (59.5)	89 (54.9)	75/97	62/89	1.13 (0.809–1.587)	0.7676	0.4647
Female	66 (40.5)	73 (45.1)	42/66	51/73	0.82 (0.547–1.241)	0.1764	0.3527
Race:							
White	97 (59.5)	99 (61.1)	75/97	77/99	0.98 (0.713–1.348)	0.4525	0.9050
Asian	51 (31.3)	44 (27.2)	31/51	22/44	1.15 (0.666–2.004)	0.6950	0.6101
Not reported	14 (8.6)	12 (7.4)	10/14	9/12	0.79 (0.317–1.945)	0.3003	0.6007
Region:							
US	10 (6.1)	10 (6.2)	8/10	7/10	1.19 (0.430–3.293)	0.6316	0.7368
EU	93 (57.1)	103 (63.6)	72/93	82/103	0.93 (0.678–1.277)	0.3284	0.6569
ROW	60 (36.8)	49 (30.2)	37/60	24/49	1.24 (0.739–2.075)	0.7912	0.4175
ECOG performance status at baseline:							
0	43 (26.4)	38 (23.5)	27/43	21/38	1.06 (0.597–1.869)	0.5752	0.8497
1	80 (49.1)	78 (48.1)	58/80	55/78	1.13 (0.783–1.639)	0.7459	0.5082
≥2	40 (24.5)	46 (28.4)	32/40	37/46	0.81 (0.503–1.314)	0.1974	0.3949
BM blasts at baseline:							
<30%	51 (31.3)	50 (30.9)	33/51	30/50	1.07 (0.653–1.757)	0.6059	0.7882
≥30%	108 (66.3)	106 (65.4)	80/108	77/106	0.97 (0.706–1.321)	0.4143	0.8285
Hematologic disease history:							
de novo	118 (72.4)	120 (74.1)	81/118	83/120	0.91 (0.670–1.239)	0.2767	0.5534
Secondary	45 (27.6)	42 (25.9)	36/45	30/42	1.22 (0.750–1.983)	0.7875	0.4250
Baseline WBC category (10^9/L):							
<10	103 (63.2)	121 (74.7)	72/103	80/121	0.99 (0.720–1.363)	0.4768	0.9536
≥10	58 (35.6)	41 (25.3)	44/58	33/41	0.82 (0.523–1.291)	0.1969	0.3938

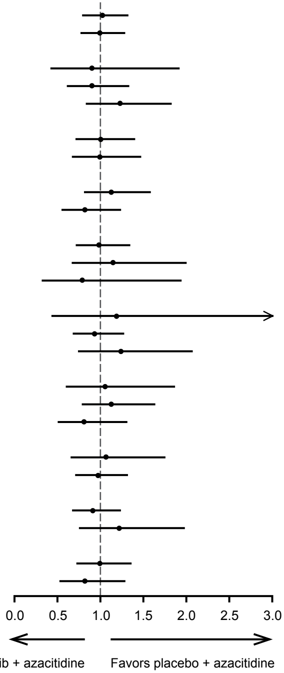

Percentages calculated based on the number of patients in the full analysis set in each treatment group.
Black or African American is removed from race categories since number of patients is too small (≤10).
BM, bone marrow; CI, confidence interval; ECOG, Eastern Cooperative Oncology Group; ELN, European LeukemiaNet; ROW, rest of world; WBC, white blood cell count

Supplementary Fig. S3 Box plots of (**A**) platelets and (**B**) neutrophils over time for all patients in the intensive study

A Platelets

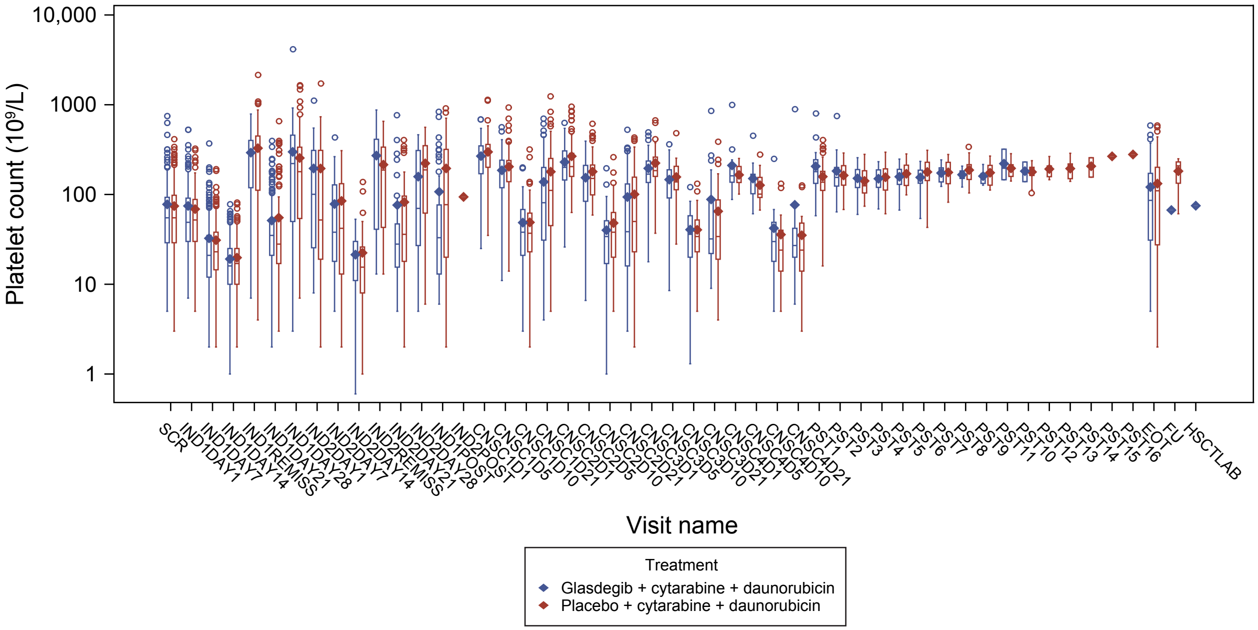

B Neutrophils

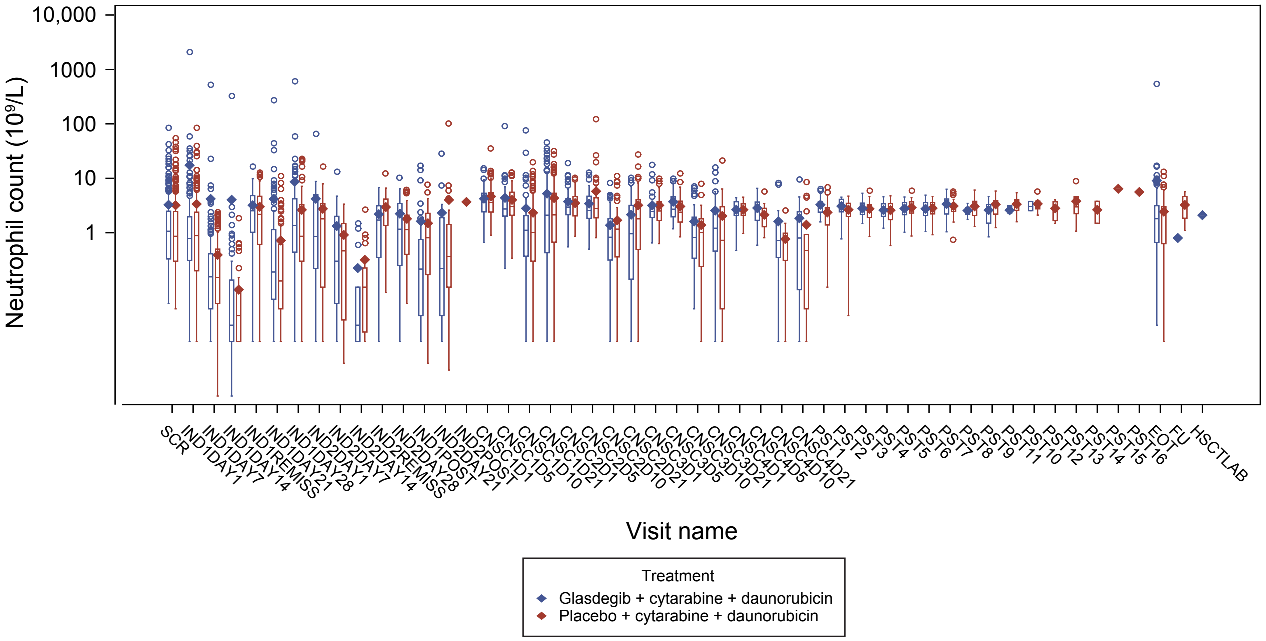

Unplanned observations during on-treatment period have been excluded from the presentation.
The mean is displayed as a diamond-filled shape, median as a horizontal line in the box interior, outliers as circles, Q1–Q3 as a box, and last point 1.5 times interquartile range as whiskers.
CNS, consolidation; C, Cycle; D, Day; EOT, end of treament; FU, follow-up; HSCTLAB, hematopoietic stem cell transplantation laboratory; IND, induction; PST, post-consolidation; Q, quartile; SCR, screening

Supplementary Fig. S4 Box plots of (**A**) platelets and (**B**) neutrophils over time for all patients in the non-intensive study

A Platelets

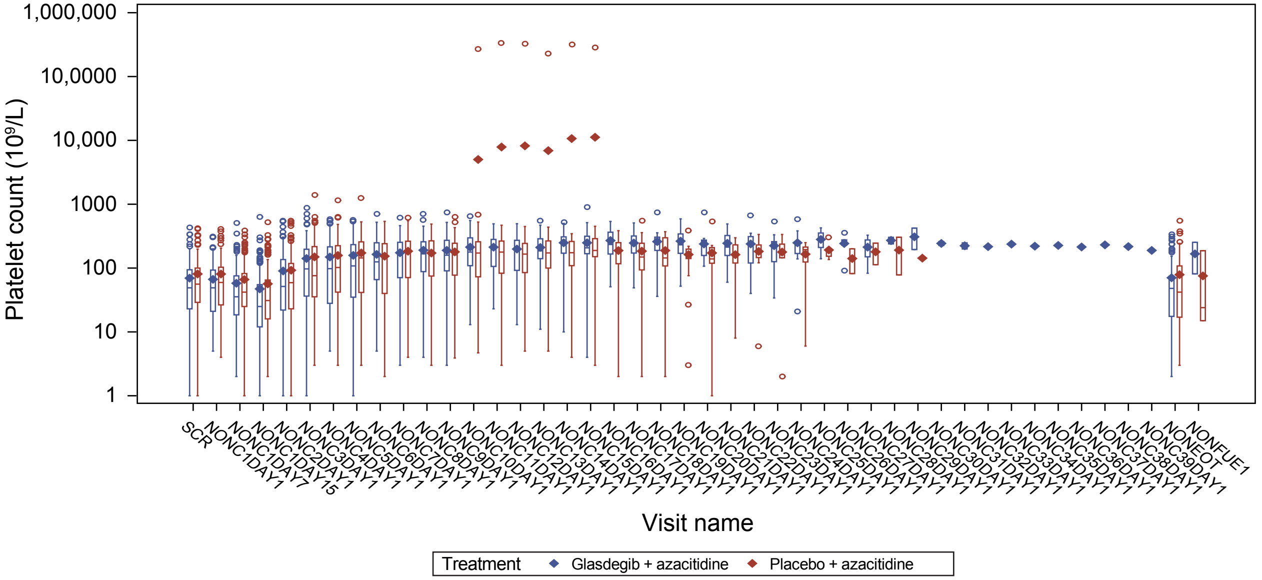

B Neutrophils

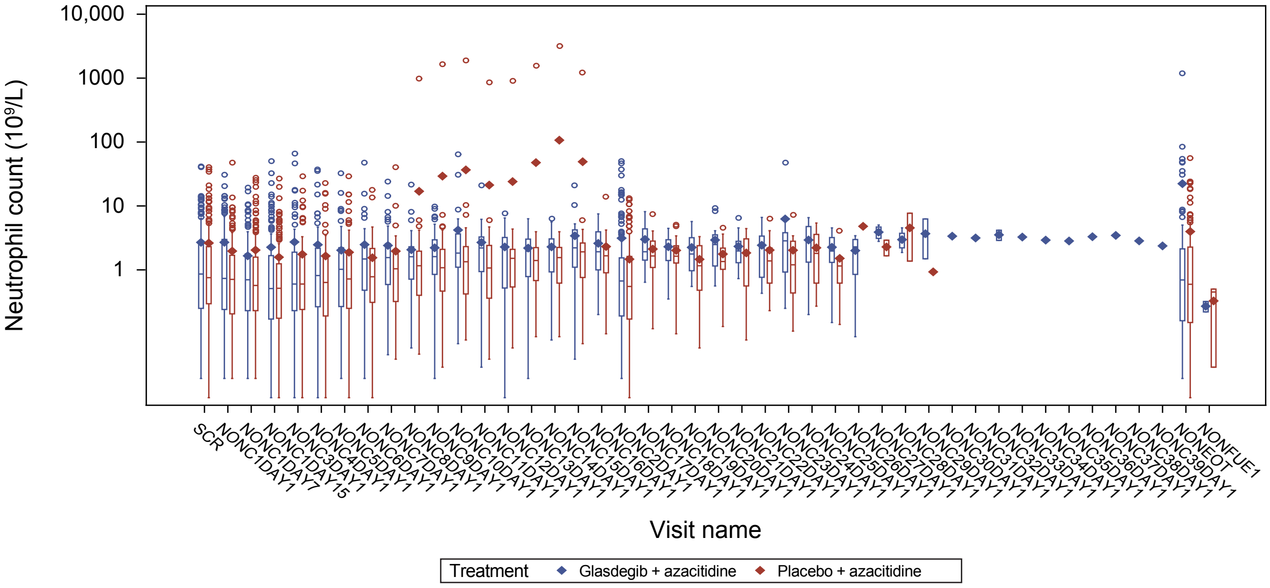

Unplanned observations during on-treatment period have been excluded from the presentation.
The mean is displayed as a diamond-filled shape, median as a horizontal line in the box interior, outliers as circles, Q1–Q3 as a box, and last point 1.5 times interquartile range as whiskers.
C, cycle; E, event; EOT, end of treament; FU, follow-up; NON, non-intensive; SCR, screening

Supplementary Fig. S5 Overall survival by ELN risk category, in patients treated with glasdegib or placebo plus cytarabine and daunorubicin in the intensive study

Subgroup	No. patients, *n* (%)		No. events/ no. patients (*N*)		HR (95% CI)	1-sided *p*-value	2-sided *p*-value
	Glasdegib + cytarabine + daunorubicin	Placebo + cytarabine + daunorubicin	Glasdegib + cytarabine + daunorubicin	Placebo + cytarabine + daunorubicin			
All participants (stratified)	198 (100.0)	201 (100.0)	89/198	86/201	0.94 (0.752–1.185)	0.3166	0.6332
All participants (unstratified)	198 (100.0)	201 (100.0)	89/198	86/201	1.02 (0.818–1.284)	0.5848	0.8303
Derived ELN risk category:							
Favorable	47 (23.7)	52 (25.9)	10/47	17/52	0.71 (0.328–1.532)	0.1904	0.3808
NPM1 mutation without *FLT3*-ITD	20 (10.1)	25 (12.4)	5/20	9/25	0.83 (0.278–2.494)	0.3717	0.7433
t(8;21)(q22;q22.1); *RUNX1-RUNX1T1*	12 (6.1)	15 (7.5)	4/12	4/15	1.41 (0.350–5.644)	0.6856	0.6289
Intermediate	79 (39.9)	70 (34.8)	37/79	21/70	1.99 (1.164–3.390)	0.9949	0.0103
Cytogenetic abnormalities not classified as favorable or adverse	67 (33.8)	63 (31.3)	34/67	23/63	1.39 (0.820–2.364)	0.8905	0.2191
Wild-type *NPM1* without *FLT3*-ITD (without adverse-risk genetic lesion)	12 (6.1)	14 (7.0)	4/12	2/14	1.95 (0.357–10.677)	0.7842	0.4316
Adverse	67 (33.8)	78 (38.8)	40/67	47/78	0.92 (0.701–1.208)	0.2771	0.5541
−17/abn(17p)	11 (5.6)	12 (6.0)	9/11	10/12	0.98 (0.398–2.431)	0.4862	0.9724
−5 or del(5q)	23 (11.6)	19 (9.5)	16/23	15/19	0.79 (0.389–1.610)	0.2587	0.5174
−7	28 (14.1)	26 (12.9)	18/28	15/26	1.07 (0.539–2.124)	0.5791	0.8418
Complex karyotype	36 (18.2)	31 (15.4)	25/36	21/31	1.00 (0.559–1.791)	0.5022	0.9956
Monosomal karyotype	9 (4.5)	13 (6.5)	6/9	10/13	0.93 (0.335–2.566)	0.4425	0.8849
Mutated *ASXL1*	6 (3.0)	15 (7.5)	3/6	9/15	0.55 (0.141–2.153)	0.1928	0.3857
Mutated *RUNX1*	9 (4.5)	14 (7.0)	5/9	10/14	0.51 (0.159–1.661)	0.1289	0.2578
Mutated *TP53*	11 (5.6)	15 (7.5)	9/11	11/15	1.22 (0.487–3.079)	0.6711	0.6578

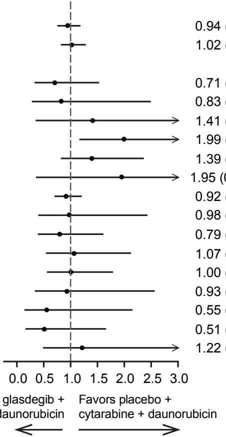

Percentages calculated based on *N*, the number of participants in the full analysis set in each treatment group.
Races (Black or African American, American Indian or Alaska Native, and Multiracial) with too small number of participants (≤10) were removed from race categories.
CI, confidence interval; ELN, European LeukemiaNet; HR, hazard ratio; ITD, internal tandem duplication

Supplementary Fig. S6 Overall survival by ELN risk category, in patients treated with glasdegib or placebo plus azacitidine in the non-intensive study

Subgroup	No. patients, *n* (%) Glasdegib + azacitidine	No. patients, *n* (%) Placebo + azacitidine	No. events/no. patients (*N*) Glasdegib + azacitidine	No. events/no. patients (*N*) Placebo + azacitidine	HR (95% CI)	1-sided *p*-value	2-sided *p*-value
All participants (stratified)	162 (100.0)	160 (100.0)	116/162	111/160	1.13 (0.918–1.396)	0.8762	0.2475
All participants (unstratified)	162 (100.0)	160 (100.0)	116/162	111/160	1.12 (0.907–1.371)	0.8488	0.3023
Derived ELN risk category:							
Favorable	27 (16.7)	19 (11.9)	15/27	12/19	0.88 (0.415–1.852)	0.3766	0.7533
NPM1 mutation without *FLT3*-ITD	12 (7.4)	11 (6.9)	8/12	6/11	0.99 (0.331–2.992)	0.4964	0.9928
Intermediate	76 (46.9)	76 (47.5)	50/76	49/76	0.87 (0.603–1.264)	0.2380	0.4760
Cytogenetic abnormalities not classified as favorable or adverse	70 (43.2)	73 (45.6)	50/70	48/73	1.14 (0.770–1.701)	0.7471	0.5058
Wild-type *NPM1* without *FLT3*-ITD (without adverse-risk genetic lesion)	15 (9.3)	6 (3.8)	11/15	1/6	5.89 (0.757–45.788)	0.9726	0.0547
Adverse	58 (35.8)	62 (38.8)	50/58	48/62	1.43 (1.09–1.876)	0.9955	0.0089
−17/abn(17p)	10 (6.2)	9 (5.6)	10/10	7/9	1.75 (0.659–4.624)	0.8716	0.2567
−5 or del(5q)	23 (14.2)	23 (14.4)	20/23	22/23	0.97 (0.527–1.777)	0.4563	0.9126
−7	21 (13.0)	21 (13.1)	19/21	18/21	0.99 (0.521–1.900)	0.4939	0.9877
Complex karyotype	27 (16.7)	30 (18.8)	25/27	24/30	1.40 (0.797–2.475)	0.8797	0.2407
Monosomal karyotype	8 (4.9)	9 (5.6)	7/8	7/9	1.97 (0.682–5.713)	0.8950	0.2100
Mutated *RUNX1*	9 (5.6)	11 (6.9)	8/9	6/11	4.20 (1.228–14.390)	0.9931	0.0138

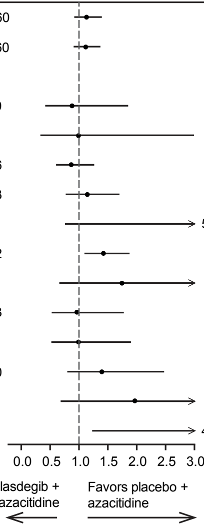

Percentages calculated based on *N*, the number of participants in the full analysis set in each treatment group.
Black or African American is removed from race categories since number of participants is too small (≤10).
CI, confidence interval; ELN, European LeukemiaNet; HR, hazard ratio; ITD, internal tandem duplication

Supplementary Fig. S7 Overall survival curves for (**A**) *GLI2* mutations in the intensive study and (**B**) *RUNX1* and (**C**) *IDH1* mutations in the non-intensive studies. Associated interaction *p*-values and the hazard ratio and *p*-value for each treatment arm shown

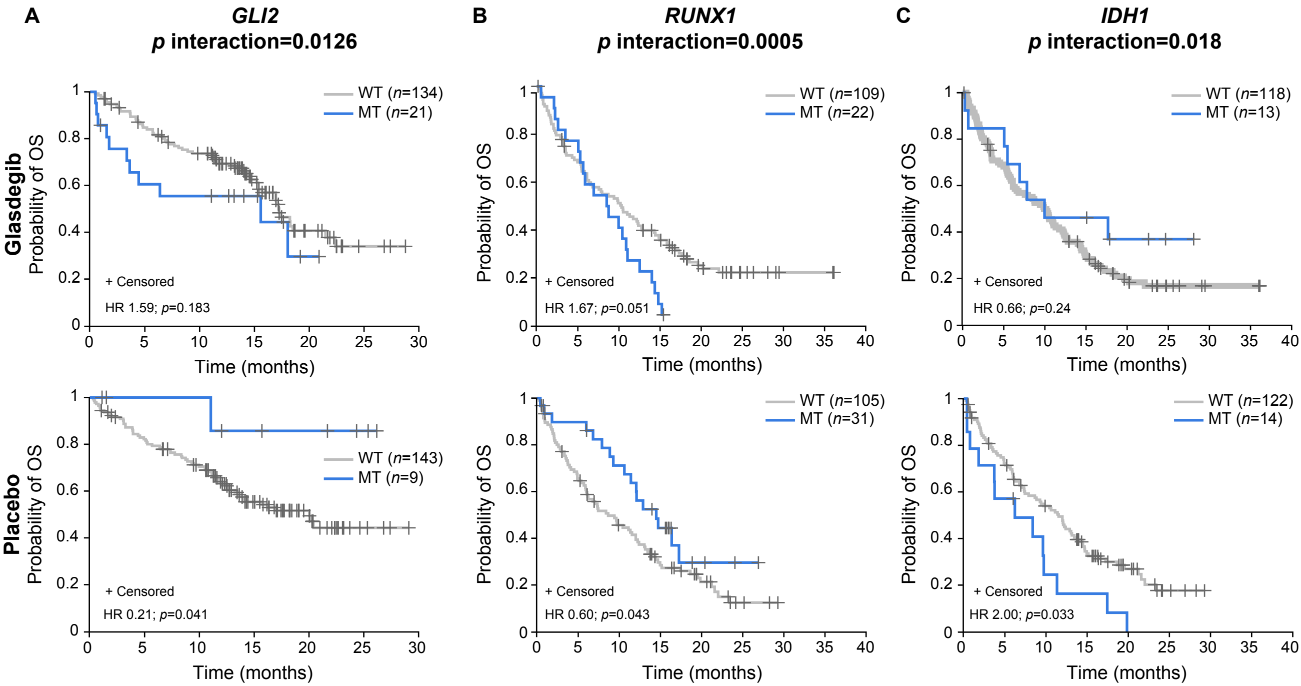

HR, hazard ratio; MT, mutation; OS, overall survival; WT, wild-type

Supplementary Fig. S8 Location of mutations in *GLI2* in the intensive study

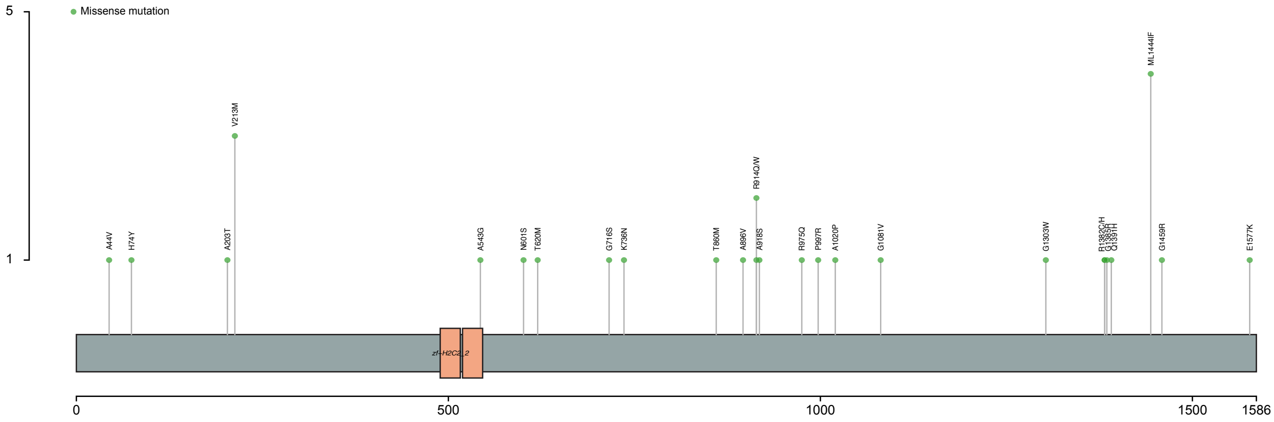

Supplement: Supplementary file 1 — Supplemental material [file 41375_2023_2001_MOESM1_ESM.pdf]
